# GC-MS Based Plasma Metabolomics for Identification of Candidate Biomarkers for Hepatocellular Carcinoma in Egyptian Cohort

**DOI:** 10.1371/journal.pone.0127299

**Published:** 2015-06-01

**Authors:** Mohammad R. Nezami Ranjbar, Yue Luo, Cristina Di Poto, Rency S. Varghese, Alessia Ferrarini, Chi Zhang, Naglaa I. Sarhan, Hanan Soliman, Mahlet G. Tadesse, Dina H. Ziada, Rabindra Roy, Habtom W. Ressom

**Affiliations:** 1 Department of Oncology, Lombardi Comprehensive Cancer Center, Georgetown University Medical Center, Washington, DC, United States of America; 2 Department of Histology and Genetics, Tanta Faculty of Medicine, Tanta University, Tanta, Egypt; 3 Department of Tropical Medicine and Infectious Diseases, Tanta Faculty of Medicine, Tanta University, Tanta, Egypt; 4 Department of Mathematics and Statistics, Georgetown University, Washington, DC, United States of America; Drexel University College of Medicine, UNITED STATES

## Abstract

This study evaluates changes in metabolite levels in hepatocellular carcinoma (HCC) cases vs. patients with liver cirrhosis by analysis of human blood plasma using gas chromatography coupled with mass spectrometry (GC-MS). Untargeted metabolomic analysis of plasma samples from participants recruited in Egypt was performed using two GC-MS platforms: a GC coupled to single quadruple mass spectrometer (GC-qMS) and a GC coupled to a time-of-flight mass spectrometer (GC-TOFMS). Analytes that showed statistically significant changes in ion intensities were selected using ANOVA models. These analytes and other candidates selected from related studies were further evaluated by targeted analysis in plasma samples from the same participants as in the untargeted metabolomic analysis. The targeted analysis was performed using the GC-qMS in selected ion monitoring (SIM) mode. The method confirmed significant changes in the levels of glutamic acid, citric acid, lactic acid, valine, isoleucine, leucine, alpha tocopherol, cholesterol, and sorbose in HCC cases vs. patients with liver cirrhosis. Specifically, our findings indicate up-regulation of metabolites involved in branched-chain amino acid (BCAA) metabolism. Although BCAAs are increasingly used as a treatment for cancer cachexia, others have shown that BCAA supplementation caused significant enhancement of tumor growth via activation of mTOR/AKT pathway, which is consistent with our results that BCAAs are up-regulated in HCC.

## Introduction

Hepatocellular carcinoma (HCC) is a type of liver cancer with high mortality rate (1-, 3-, and 5-year survival rates of 49%, 19%, and <10%, respectively) [1]. Malignant conversion of cirrhosis to HCC is often fatal in part because adequate biomarkers are not available for diagnosis of HCC at the early stage. Alpha-fetoprotein (AFP), the serologic biomarker for HCC in current use, lacks the desired sensitivity [2,3]. Therefore, more potent biomarkers are needed for detection of HCC at its early stage when it can be intervened more effectively. The goal of this study is to identify potential metabolic biomarkers by evaluating the metabolite levels in plasma samples from HCC cases and patients with liver cirrhosis.

Metabolomics is a rapidly evolving tool to study small molecules (molecular weight <1800Da) that define the metabolic status of a biological system. It has been applied extensively to discover biomarkers for liver disease diagnosis and to better understand the pathophysiology [4–6]. Various metabolomics studies have led to the identification of significant differences of bile acids, phospholipids and fatty acids, along with alteration in glycolysis pathway, urea cycle and methionine metabolism, in blood, urine and fecal samples of patients with HCC compared with benign liver tumor or healthy subjects [7–13].

A number of candidate biomarkers for HCC have been discovered by using liquid chromatography coupled to mass spectrometry (LC-MS) for analysis of metabolites in human biological fluids and tissues. For example, glycodeoxycholate, deoxycholate 3-sulfate, and bilirubin were identified in tissues as candidates distinguishing HCC vs. cirrhosis [10]. Also, valine and glutamine pathways were found up-regulated in liver tissues from HCC vs. those from cirrhotic controls [14]. Citric acid was also found to be significantly different between HCC cases and cirrhotic controls in serum [15]. We previously observed down-regulation of bile acids and up-regulation of phospholipids and amino acids in HCC cases vs. cirrhotic controls through metabolomics analysis of sera by LC-MS [16–18]. Specifically, we observed down-regulation of long chain carnitine, oleoyl carnitine, palmitoyl carnitine, and linoelaidyl carnitine in HCC patients compared with cirrhotic controls.

It is widely accepted that not a single technique is feasible to investigate the whole range of chemical species and concentration levels that characterize the human metabolome. Gas chromatography coupled to mass spectrometry (GC-MS) has been used as a complementary approach to LC-MS to increase the metabolome coverage or to verify the identification of the potential biomarkers found by LC-MS [10,19–21]. For example, GC-MS has enabled the detection of compounds such as intermediates of Krebs cycle and glycolysis pathways, which have been reported to be consistently altered in cancer metabolism [22]. Also, analysis of urine samples by GC-MS has led to the identification of ethanolamine, lactic acid, acotinic acid, phenylalanine, and ribose as potential markers distinguishing HCC from cirrhosis [23]. Similarly, by comparing plasma samples from HCC and healthy controls by GC-MS, several metabolites were found significant including butanoic acid, ethanimidic acid, glycerol, isoleucine, valine, aminomalonic acid, D-erythrose, hexadecanoic acid, octadecanoic acid, and octadecadienoic acid [24]. Furthermore, GC-MS was used in a targeted analysis to quantitatively evaluate metabolites in plasma samples that were found statistically significant between HCC and cirrhosis by LC-MS [10].

In this study, we used GC-MS to analyze plasma samples from 40 HCC cases and 49 patients with liver cirrhosis recruited in Egypt. Specifically, we performed untargeted metabolomic analysis of the plasma samples using two GC-MS platforms: an Agilent GC coupled with a single quadrupole mass spectrometer (GC-qMS) and an Agilent GC coupled to a LECO TOF mass spectrometer (GC-TOFMS). We took advantage of the combined information from the two different mass analyzers and software tools utilized for peak deconvolution to help verify the detection of analytes. Our experimental design included chromatogram quality assessment, mass accuracy and resolution check, adequate quality control (QC) runs, system cleanup, and column conditioning. The sample preparation and data acquisition were performed in multiple batches to address the technical limitation on the number of samples that can be analyzed at once. Following data processing by commercial and open source software tools, Fiehn and NIST libraries were used for metabolite identification. Two-way analysis of variance (ANOVA) models were then used for selection of analytes with significant differences in ion intensities between HCC cases and cirrhotic controls, accounting for the batch effect. The analytes selected by the ANOVA model and other candidates from related studies were further evaluated by targeted analysis in the same plasma samples, using GC-qMS in selected ion monitoring (SIM) mode. The results of the targeted analysis confirmed the significance of nine analytes as candidate biomarkers for HCC. Finally, we performed pathway analysis by combining these nine analytes with other significant metabolites we previously identified by LC-MS based analysis of sera from the same participants [25].

## Materials and Methods

### Materials

Deuterium-labeled internal standards were purchased from CDN isotopes. These include L-phenyl-d5-alanine-2,3,3,-d3 (D-1241), L-glutamic-2,3,3,4,4-d5 acid (D-899), Tyrosine-d2 (D-1611), and L-alanine-2,3,3,3-d4 (D-1488). Myristic acid–d27 (366889), Alkane standard mixture (68281), fatty acid methyl ester standards (FAMEs), C8 (260673), C9 (245895), C10 (299030), C12 (234591), C14 (P5177), C16 (P5177), C18 (S5376), C20 (10941), C22 (11940), C24 (87115), C26 (H6389), C28 (74701), except for the C30, were purchased from TCI chemicals (T0812), while methoxyamine hydrochloride (226904) and pyridine (360570) were purchased from Sigma Aldrich. MSTFA (TS-48910) was purchased from Thermo Scientific. HPLC grade 2-propanol, acetonitrile and water were used for metabolites extraction. Helium was purchased from Robert Oxygen.

### Study cohort

Adult patients were recruited from the outpatient clinics and inpatient wards of the Tanta University Hospital in Tanta, Egypt. The participants consist of 89 subjects (40 HCC cases and 49 patients with liver cirrhosis). The protocol of the study was approved by the ethical committee at Tanta University. Detailed characteristics of the patient populations are provided in Table 1. Through peripheral venepuncture, single blood sample was drawn into 10 mL BD Vacutainer sterile vacuum tubes in presence of EDTA anticoagulant. The blood was immediately centrifuged at 1000g for 10 min at room temperature. The plasma supernatant was carefully collected and centrifuged at 2500g for 10 min at room temperature. After aliquoting, plasma was kept frozen at −80°C until use. Primary tubes and plasma aliquots were labeled using anonymous confidential code numbers with no personal identifiers. Identification codes were cross-referenced with clinical information in a pass code protected computer system.

**Table 1:** Characteristics of the study cohort.

		HCC (*n* = 40)	Cirrhosis (*n* = 49)	*p*-value
**Age**	Mean (SD)	53.2 (3.9)	53.8 (7.6)	0.3530
**BMI**	Mean (SD)	24.9 (3.1)	24.5 (4.4)	0.6513
**Gender**	Male	77.5%	67.3%	0.3474
**HCV serology**	HCV Ab+	100.0%	100.0%	1.0000
**HBV serology**	HBsAg+	0.0%	6.1%	0.2492
**MELD**	Mean (SD)	18.6 (7.7)	18.9 (7.1)	0.1328
	MELD ≤ 10	20.0%	12.2%	0.3863
**Child-Pugh grade**	A	15.0%	0%	
	B	47.5%	46.9%	0.0117
	C	37.5%	53.1%	
**AFP**	Median (IQR)	275.9 (1244.3)		
**HCC stage**	Stage I	72.5%		
	Stage II	15.0%		
	Stage III	5.0%		
	Unknown	7.5%		

### Ethics Statement

This study used non-identifiable plasma samples collected at Tanta University, Tanta, Egypt following approval of the study protocol by the Tanta University Ethical Committee (Protocol Approval # 113/03/10). Adult patients with HCC or cirrhosis were prospectively recruited from the outpatient clinics and inpatient wards of the Department of Tropical Medicine and Infectious Diseases at Tanta University Hospital, Tanta, Egypt. All participating patients provided written informed consent before taking part in the study. Following the participant’s informed consent signature and enrollment, the subject’s blood was drawn, processed, and stored. Frozen specimens were transported to the U.S. on dry ice via overnight shipping. Primary tubes and plasma aliquots were labeled using anonymous confidential code numbers with no personal identifiers.

### Experimental design

**Fig 1:**
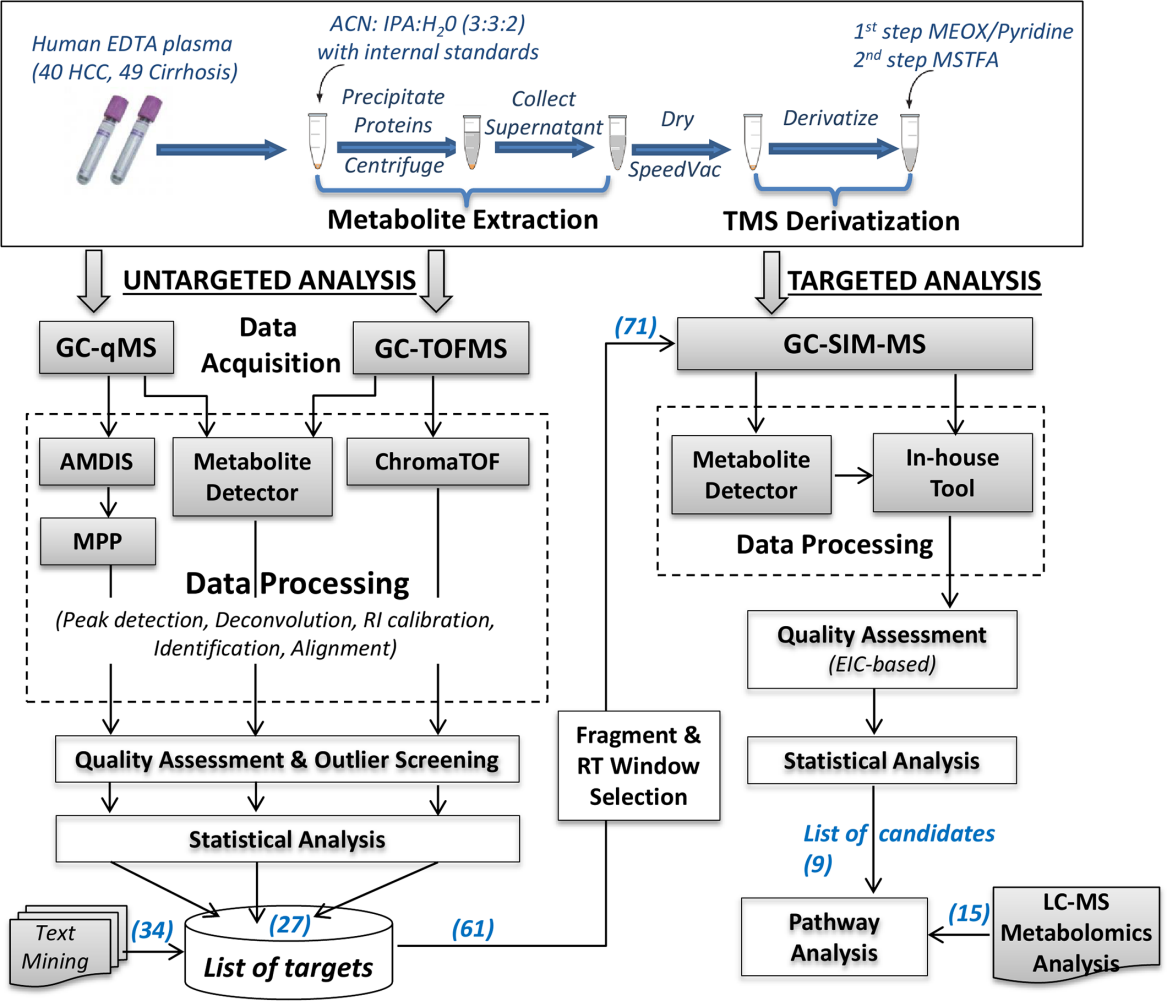
Workflow of our GC-MS based untargeted metabolomic and targeted analyses for biomarker discovery. The number of candidate metabolites analyzed at specific steps is shown in parenthesis.

Fig 1 shows the overall experimental design including sample preparation, data acquisition, data processing, and statistical methods for untargeted and targeted analyses. The samples were split into batches with balanced proportions of cases and controls in each batch to allow adequate time intervals between batches for sample preparation and for calibration of the GC-MS instruments. Due to the length of the chromatographic separation, the untargeted metabolomic analysis by GC-qMS involved four batches (B1-B4), while only two batches were considered for GC-TOFMS by merging B1 & B2 in the first batch and B3 & B4 in the second batch. In addition to cases and controls, a pooled QC sample was analyzed in each batch by taking an equal volume from each prepared biological sample within the batch. The QC was run multiple times at the beginning of each batch, in between runs, and at the end of each batch. The QCs at the beginning were used for column conditioning. The data from the remaining QC runs were used for quality assessment. Also, a “blank” sample containing only the derivatization reagents was run at the beginning of each batch to assess the background ions introduced by sample derivatization. A mixture of retention index (RI) standards was spiked in each sample for RI calibration. The analysis order of the samples is provided in S1 Table. Also more details about the RI standards and quality assessment approach can be found in S1 Document.

### Metabolite extraction

Plasma metabolites were extracted by adding 1mL of working solution composed of acetonitrile, isopropanol, and water (3:3:2) containing isotope-labeled internal standards at a concentration of 1.25μg/mL (tyrosine-3,3-d2, glutamic acid-2,3,3,4,4-d5, alanine-2,3,3,3-d4, phenylalanine-phenyl-d5-2,3,3,-d3, myristic acid d27) to 30μL of plasma. After vortexing, samples were centrifuged at 14,500 *g* for 15 minutes at room temperature. The supernatant was then divided into two, 460μL each, for analysis by each of the two GC-MS systems. Each supernatant was then concentrated to dryness in speedvac. The dried samples were kept at -20°C until derivatization prior to analysis by GC-MS.

### Derivatization

We derivatized the dried samples in each batch prior to injection following a two-stage process of oximation followed by trimethylsilylation (TMS) [26,27]. Briefly, 20μL of a 20mg/mL methoxyamine hydrochloride in pyridine was added to the dried extracts, vortexed and incubated at 80°C for 20 minutes. After returning the samples at room temperature, 91μL of MSTFA + RI standards was added, vortexed and incubated at 80°C for 20 minutes. Samples were then centrifuged at 14,500rpm for 15 minutes, and 60μL of the supernatant was transferred into 250μL clear glass autosampler vials.

### Acquisition of GC-MS data by untargeted method

Metabolites extracted from plasma samples were analyzed by using both the GC-qMS (Agilent 5975C MSD coupled to an Agilent 7890A GC) and GC-TOFMS (LECO Pegasus TOF coupled to an Agilent 7890A GC) systems both equipped with an Electron Impact ionization source. For the GC-qMS, Agilent J&W DB-5MS column (30m × 0.25mm × 0.25μm film 95% dimethyl/ 5% diphenyl polysiloxane) + 10m Duragard Capillary column was installed and calibrated. Prior to sample analysis, the GC system was locked by performing retention time locking (RTL) of myristic acid d27 at 16.727 minutes. The samples were injected in splitless mode, with the injection port held at 250°C. The initial oven temperature was held at 60°C for one minute and then ramped at 10°C/min to 325°C and held for 10 minutes. The post run is one minute to allow the oven cool down to 60°C. MSD transfer line was held at 290°C, ion source at 250°C and the mass analyzer at 150°C. The GC-qMS data were acquired in 37.5 minutes with 5.9 minute solvent delay at normal scan rate in the mass range 50–600Da.

For the GC-TOFMS system, Agilent HP-5 column (30m × 0.32mm id × 0.25μm film 95% dimethyl 5% diphenyl polysiloxane) was used. 99.9% pure Helium was set at a constant flow of 1.5mL/min. The oven temperature was held constant at 70°C for 4 minutes and then ramped at 20°C/min to 300°C at which it was held constant for 5 minutes. The transfer line temperature between the GC and the MS was set to 225°C. The ion source temperature was set at 200°C. After 5 minutes of solvent delay, filament was turned on and mass spectra were acquired over the range of m/z of 50–600Da at an acquisition rate of 20 scans per second. The detector was operated in the range of 1400–1600V with an optimized voltage offset of 200V. Recording ended after 19.5 minutes.

### Analysis of GC-MS data acquired by untargeted method

The GC-TOFMS data were analyzed using the LECO ChromaTOF software. The GC-qMS data were processed by the Automated Mass Spectral Deconvolution and Identification System (AMDIS) [28] for peak detection, deconvolution, and metabolite identification. The results were then imported into Mass Profiler Professional (MPP) for alignment and statistical analysis. In addition, both the GC-TOFMS and GC-qMS data were analyzed by MetaboliteDetector [29], which allows the utilization of RI values for alignment using the RI values calculated for every detected analyte based on RI calibration that were performed for each batch separately. The calculated RI values were also used for narrowing the library search results, thereby reducing the number of false putative identifications. Also, MetaboliteDetector enables us to select the top three fragments based on the quality of their EICs. Putative metabolite identifications were determined based on spectral matching using the Fiehn and NIST libraries. The Fiehn library includes spectra only from TMS compounds. To reduce the false discovery rate in the metabolite identification process using the NIST library, we extracted all compounds acquired by TMS derivatization (around 5,000 compounds with more than 22,000 spectra).

Prior to statistical analysis, raw intensities were log-transformed to make the component intensity distributions more comparable with a normal probability density function. Based on the number of measurements available for each feature across the runs, we used three different steps to evaluate the statistical significance comparing cases and controls. If a peak is found in most of the runs from one group and is missing completely in the other group, it is considered as a potential candidate without any further statistical hypothesis testing. If a peak is present in both groups, but missing in many runs, a logistic regression model is used to find the difference between cases and controls and significance is estimated based on the calculated p-values. The remaining peaks were analyzed by using a two-way ANOVA model. To control the false discovery rate (FDR), we calculated q-values using the Benjamini-Hochberg method. Analytes were selected based on two criteria (consistent mean fold change direction in all batches and *q* < 0.1), i.e., analytes with significant group effect but insignificant batch-group interaction were selected. The mean fold change was calculated for each batch separately based on the cases and controls within the batch. Also the batch-group interaction term was included to avoid overestimation of the error variance as it can increase the number of false negatives.

### Acquisition of GC-SIM-MS data by targeted method

Targeted quantification was performed in SIM mode by using the GC-qMS platform. The methods for sample preparation, GC separation, injection, and RI calibration were the same as the untargeted metabolomic analysis by GC-qMS described in previous sections. The targets for this analysis included analytes that displayed statistically significant differences between cases and controls in our untargeted analysis and other related studies. For each analyte, four ions were selected based on their specificity and intensity, with one used as a quantifier for intensity calculation and others used as qualifiers for confirmation. The fragments were selected based on the uniqueness across co-eluting analytes and their relative intensity compared to the base peak in the spectrum. Time segments are set up to allow at least 10msec dwell time for each ion monitored.

### Analysis of GC-SIM-MS data acquired by targeted method

We used MetaboliteDetector to find the retention time (RT) values for a subset of the targets with relatively high similarity scores. These RT values were compared against those in the Fiehn library to estimate the difference between expected and observed elution times. Following that, we used an in-house tool to extract the EIC guided by the estimated RT from the previous step. The algorithm uses an RT window centered at the expected elution time of the analyte of interest and searches the neighborhood area for all detected peaks at monitored masses. The quantifier fragment is used to perform the search and all qualifiers peaks are found based on the location of the quantifier peak. Following smoothing of the EICs and baseline correction, the peak width and the area under the curve (AUC) of the EICs are calculated. Finally, a similarity score is calculated based on the expected SIM spectra from the library to verify the identification. Since only four fragments were monitored per analyte in the SIM mode, we used a more restrictive approach to calculate the spectral matching similarity score [30]. Specifically, a mixed measure is used based on two scores: (1) weighted dot product; (2) average pairwise ratios between fragments. Also, we checked each EIC by visual inspection to avoid identification errors.

## Results

### Analytes selected by untargeted method

We analyzed metabolites in plasma samples from 89 patients (40 cases and 49 controls) by untargeted analysis using both the GC-qMS and GC-TOFMS systems. Considering the run time per sample by these instruments (37.5min and 19.5min for GC-qMS and GC-TOFMS respectively), we analyzed the patient samples, blanks, QCs, and RI runs by GC-qMS in four batches and by GC-TOFMS in two batches.

We excluded three runs from the GC-qMS data due to significant inconsistency of their total ion chromatogram (TIC) compared to other runs. We did not find any outliers in the GC-TOFMS data. All five spiked deuterated internal standards (IS) were detected in almost all runs; only 3% of the total number of ISs expected in all runs combined was missed. The coefficient of variation (CV) of ISs ranged from 0.7% to 3.7% based on their log transformed intensity. Also, we used the ISs and the FAMEs RI standards to evaluate the RT shift. We observed an RT shift of less than 5 and 3 seconds for GC-qMS and GC-TOFMS data, respectively. We observed the same trend for all of the analytes in QC runs as those of the ISs.

We detected a total of 621 analytes in the GC-qMS data and 780 analytes in the GC-TOFMS data. Using the Fiehn and NIST libraries, about 32% and 35% of the analytes were identified using similarity score thresholds of 60 out of 100 and 750 out of 1000, respectively. S1 Fig shows several examples of spectral matching, where the fragmentation patterns of reference spectra are compared with that of an analyte measured in our samples. Following alignment of the detected analytes, we evaluated the CV on the basis of the QC runs. We observed an average CV of 4.1% (± 2.8%) and 3.9% (± 2.5%) for the GC-qMS and GC-TOFMS data, respectively. Although we used log transform to avoid extreme high and low values for the ion intensities, there were still some outliers in the data that can mislead the estimation of the statistical significance. Also, for some analytes, there were a considerable number of missing values. Therefore, by carefully examining the distributions of the log-transformed intensities, we filtered out unreliable analytes.

Through ANOVA models, we selected analytes with significant difference between cases and controls. We found 14 analytes from GC-qMS and 19 from GC-TOFMS data with q-values < 0.1 and with consistent fold change direction in all batches. There were three overlapping analytes between the two platforms leading to 30 unique analytes, of which, 27 identified compounds were used for the targeted analysis. Also, we included 34 analytes found significant in other related studies along with five internal standards. Since we observed more than one analyte, either with different number of TMS or as isomer forms, for some of compounds identified in the untargeted analysis, we included 10 alternative forms in our targeted analysis. For example, if we observed L-valine 2 as statistically significant, we included L-valine 1 in the list. Thus, a total of 71 (27+34+10) analytes were analyzed by SIM in the same 89 plasma samples previously analyzed by untargeted method. A subset of these analytes is provided in Table 2 along with the number of TMS groups for each analyte (the complete list can be found in S2 Table). In addition to one quantifier and two qualifier fragments, we monitored the fragment with molecular mass of 73Da as a qualifier for all 71 targets. Also, we acquired full scan GC-qMS data from pooled samples in between the experimental samples to facilitate the estimation of the RT values for the analytes of interest by spectral matching using the complete fragmentation pattern.

**Table 2:** List of nine analytes confirmed by targeted analysis with their expected retention time and molecular weights for quantifier (M1) and qualifier fragments (M2-M3). Fragment with a molecular weight of 73 was also monitored by default for all the analytes.

Name	KEGG ID	Fiehn Index	# of TMS*	RT (min)	M1	M2	M3
glutamic acid	C00025	33032	3	14.40	246	128	147
alpha tocopherol	C02477	2116	1	27.38	502	236	237
valine	C00183	6287	2	9.25	144	145	218
lactic acid	C00186	107689	2	7.00	117	147	191
citric Acid	C00158	311	4	16.61	273	147	274
sorbose	C00247	1101	5	17.10	103	147	217
cholesterol	C00187	304	1	27.55	75	129	329
leucine	C01933	21236	1	8.80	86	75	87
isoleucine	C00407	791	1	8.58	86	69	75

* Number of TMS in Fiehn library

### Significant analytes confirmed by targeted analysis

**Fig 2:**
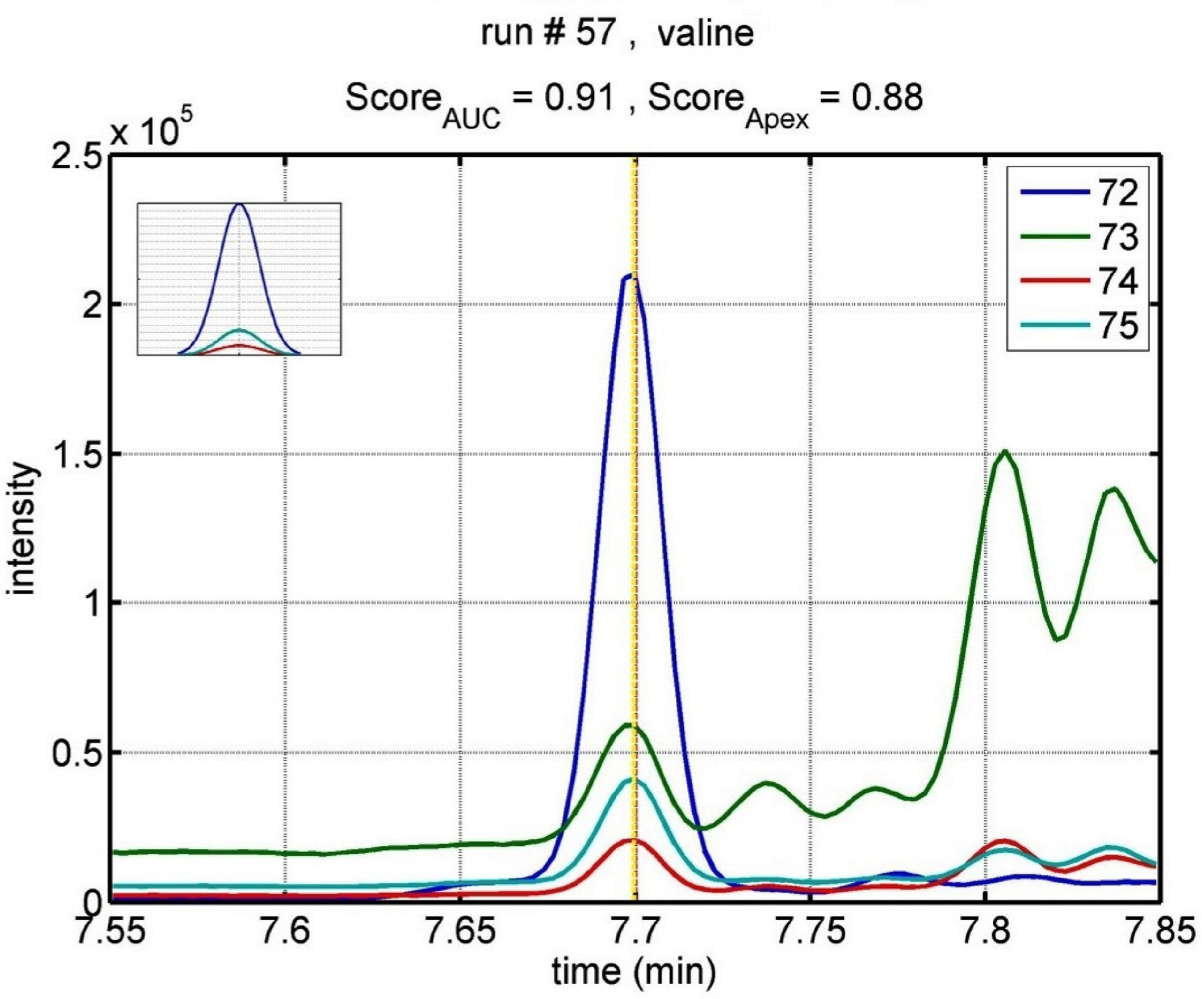
Example of a retrieved EIC for valine. The inset in the top left shows the expected ratios for the fragments based on the library to guide the visual inspection. The doted vertical lines show the expected and estimated elution time of the analyte. Although, the background signal of 73 from other compounds is reflected in the apex score, its impact on the AUC is diminished by baseline correction.

Analysis of the GC-SIM-MS data by MetaboliteDetector found the RT values for 37 metabolites out of 71. These RT values were used as an initial value to obtain the EIC-based intensities. For the remaining 34 metabolites, we used expected the RT values from the Fiehn library as the initial value. By using our in-house tool that adjusts RT points iteratively, we detected 67 out of 71 analytes with mixed similarity scores greater than 0.7 with less than 1% of missing values. Fig 2 shows an example EIC of valine 1 retrieved using our in-house tool and the mixed similarity scores based on AUC and peak apex. The apex-based score helps to avoid misidentification when co-eluting analytes are present. Statistical analysis of the 67 analytes identified nine with significant differences in ion intensities between cases and controls. Also, the fold changes for these analytes were consistent with the results from the untargeted metabolomic analysis acquired by GC-qMS and GC-TOFMS platforms.

Table 3 presents a list of significant analytes from both platforms in the untargeted analysis and those that have been confirmed by targeted analysis along with their p-values, q-values, average fold changes based across the batches, and references in which the candidates were previously reported. The table presents only those analytes that showed consistent fold change direction in the majority of the batches (*i*.*e*., 3 out of 4 in the GC-qMS analysis or 2 out of 2 in the GC-TOFMS analysis). Putative metabolites IDs are provided when available. Unique mass and RT values are provided for the unidentified analytes. Moreover, the top hits for each metabolite have been reviewed subjectively based on similarity score and chemical properties of the compounds to ensure the quality of identification process.

**Table 3:** Metabolites found relevant by untargeted and targeted analyses.

Putative ID Name	Fiehn	NIST	Platform	p-value*	q-value	Fold change
glutamic acid^a^ ^,^ ^b^	✓	✓	GC-TOFMS	4.9E-7	4.5E-5	1.1
	✓		GC-qMS	0.0204	0.3305	1.1
	✓		GC-SIM-MS	5.5E-8	N/A	1.9
alpha tocopherol	✓	✓	GC-TOFMS	0.0095	0.1725	1.1
			GC-SIM-MS	0.0012	N/A	1.5
valine^c^ ^,^ ^d^	✓	✓	GC-TOFMS	0.0124	0.2039	1.1
	✓	✓	GC-qMS	0.0104	0.3090	1.2
	✓		GC-SIM-MS	0.0033	N/A	1.5
lactic acid^e^	✓		GC-qMS	0.0212	0.3170	-1.1
	✓		GC-SIM-MS	0.0028	N/A	-1.3
citric acid^f^	✓	✓	GC-TOFMS	0.0070	0.1633	-1.1
	✓	✓	GC-qMS	0.0007	0.0774	-1.1
	✓		GC-SIM-MS	0.0095	N/A	-1.3
sorbose	✓	✓	GC-qMS	0.0040	0.1578	-1.2
	✓		GC-SIM-MS	0.0132	N/A	-2.4
leucine^d^	✓		GC-SIM-MS	0.0186	N/A	1.6
isoleucine^c^	✓	✓	GC-TOFMS	0.0620	0.4845	1.5
	✓		GC-SIM-MS	0.0423	N/A	1.5
cholesterol	✓	✓	GC-TOFMS	0.0164	0.2351	1.1
	✓		GC-SIM-MS	0.0355	N/A	1.1
Unidentified^†^ (UM 73; RT 1594)			GC-qMS	0.0001	0.0029	2.7
Unidentified^†^ (UM 232; RT 808)			GC-TOFMS	0.0001	0.0071	1.1

* The p-values are from ANOVA for the untargeted analysis (GC-qMS/GC-TOFMS) and one-tailed test for the targeted analysis (GC-SIM-MS) assuming that the direction of change (increase or decrease in metabolite level) is known from the results of the untargeted analysis.

† No identification based on the criteria we used to match against the library (UM = unique mass, RT = retention time in seconds)

^a^ HCC cases vs. normal controls [14].

^b^ Glutamic acid transporter overexpressed in HCC tissues compared to adjacent normal tissues using mRNA analysis [31].

^c^ Up-regulated in HCC vs. normal by LC-MS based analysis of tissues [14].

^d^ Up-regulated in HCC vs. normal serum by GC-MS based analysis of sera [24].

^e^ Down-regulated in HCC vs. normal by analysis of urine samples [23].

^f^ Down-regulated in HCC vs. cirrhosis by NMR and LC-MS based analyses [15].

We performed cross comparison between the GC-qMS and GC-TOFMS platforms. For each platform, we found the overlapping statistically significant ions based on available information such as chemical name and CAS number. If an analyte was found statistically significant by one platform, we checked its significance and fold change in the data from the other platform to see if the direction of the change is consistent. For unidentified analytes, we created a library by extracting true spectra from the raw data and searched them against those measured by the other platform. The true spectra were determined by searching for those runs with the highest purity, *i*.*e*., those with the least overlapping/co-eluting peaks. Also, by comparing every pair of extracted spectra of unidentified components from both instruments, we searched for overlapping unidentified analytes. The spectra of these unknown analytes are included in S3 Table.

### Verification of the identities of significant metabolites

To verify the identity of the metabolites found statistically significant in our targeted analysis, we ran authentic standards side by side with our samples. S1 Fig shows the spectral matching between standard compounds and plasma metabolites. Also, details of the confirmation analysis are described in, the caption of the figure. By comparing the fragmentation patterns of the standards against those from our samples, we confirmed the identities of valine, isoleucine, leucine, alpha tocopherol, citric acid, lactic acid, glutamic acid, and cholesterol. While we confirmed that one of the significant metabolites belongs to the class of furanose sugars, we were unable to determine its identity with certainty. However, based on RIs for two standards (sorbose and tagatose), we determined sorbose as the likely identification.

## Discussion

**Fig 3:**
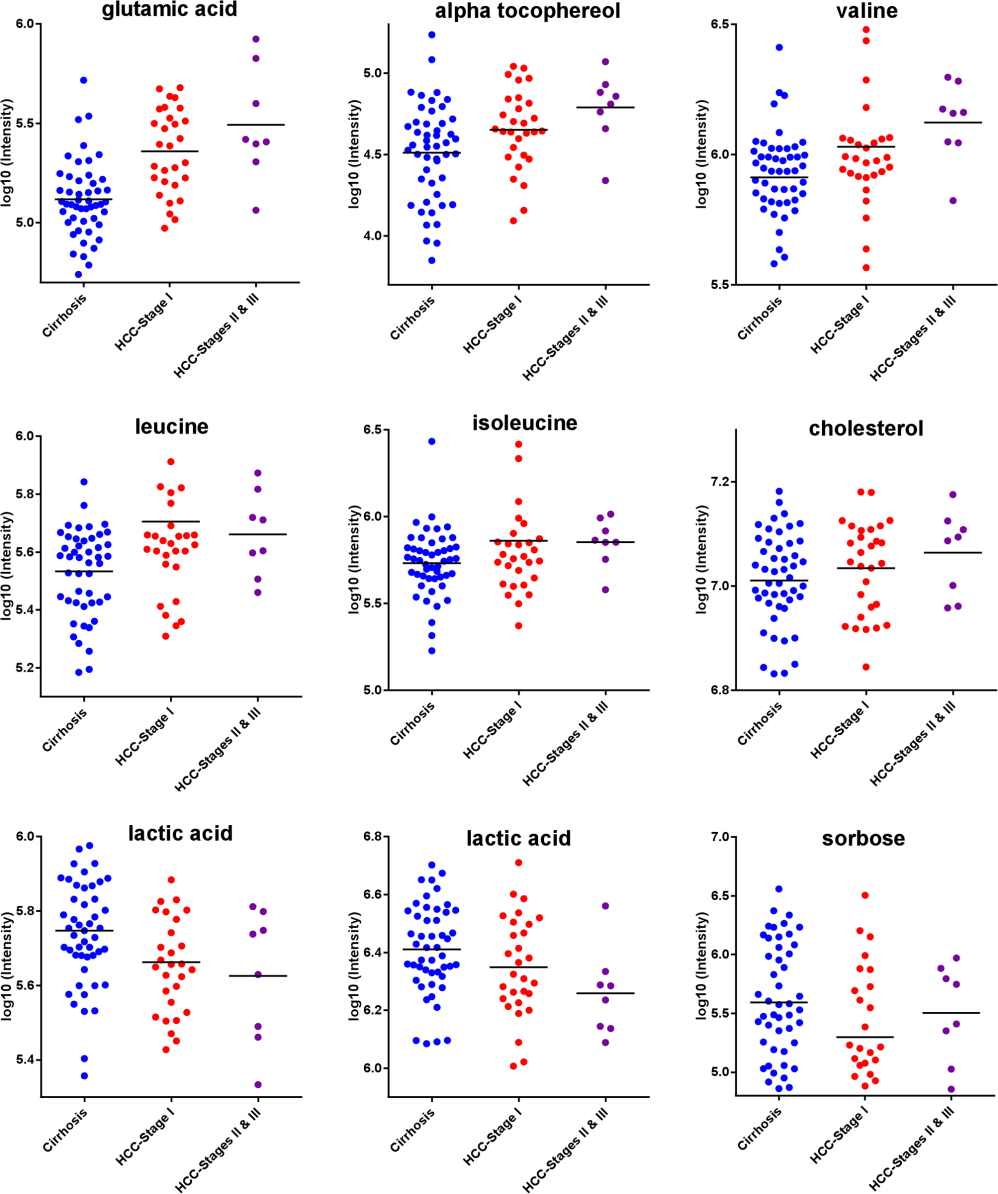
Metabolites with significant changes in their levels in HCC vs. cirrhosis based on targeted analysis of plasma by GC-SIM-MS. The metabolites in the top two panels show increasing trend with the progression of HCC. The metabolites in the bottom panel are down-regulated in HCC vs. cirrhosis. While lactic acid and citric acid show decreasing trend with the progression of HCC, sorbose is down-regulated overall in HCC vs. cirrhosis.

**Fig 4:**
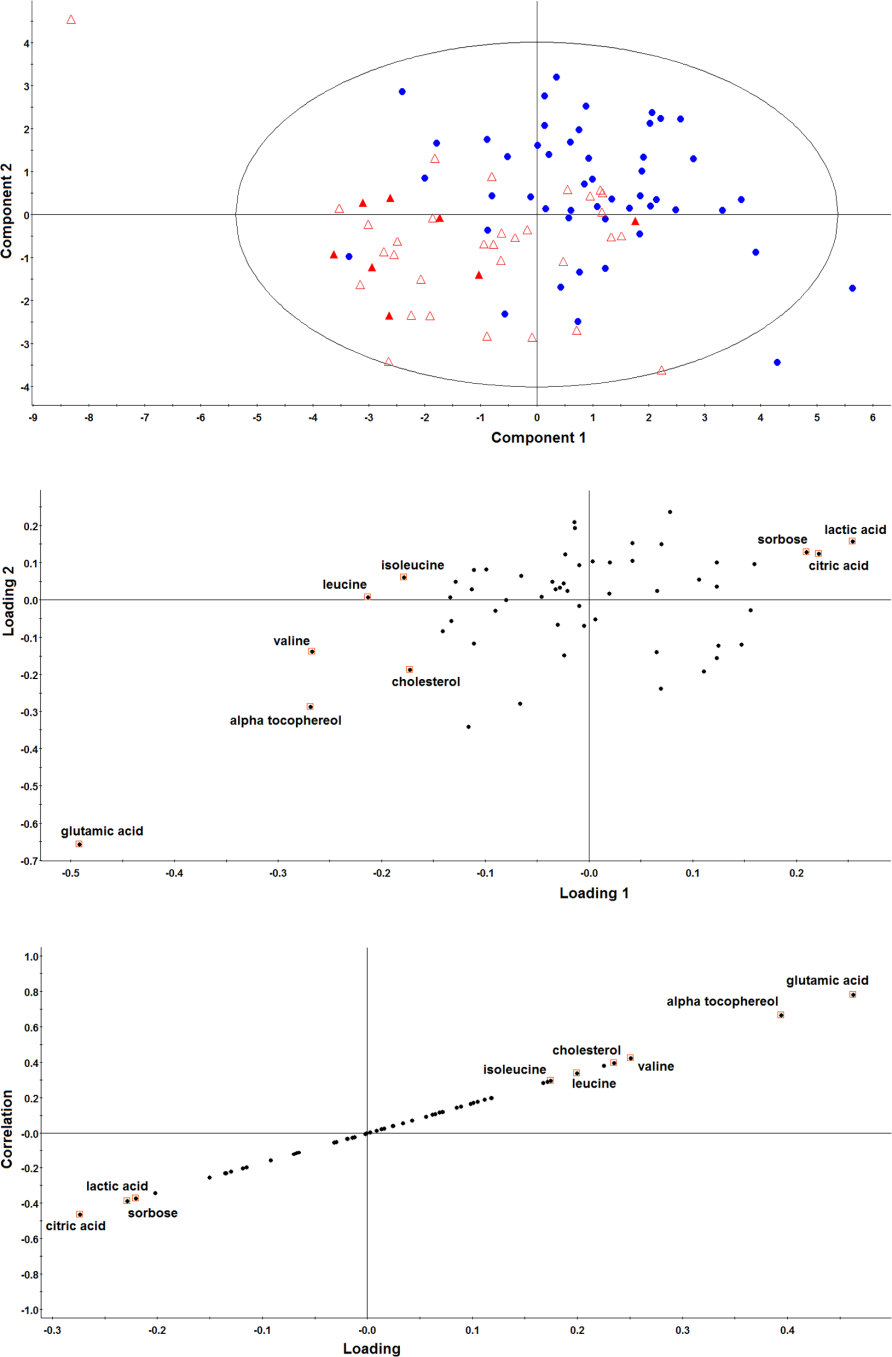
Evaluation of the metabolites from targeted analysis using PLS-DA and OPLS-DA. A: Score plot obtained by PLS-DA with HCC cases labeled by red triangles and patients with liver cirrhosis by blue dots. Stage II & III HCC cases are labeled with solid triangles. B: Loading plot from PLS-DA. C: S-plot obtained by OPLS-DA. The nine metabolites previously selected by univariate analysis are highlighted in B and C.

In summary, our results show that glutamic acid, valine, leucine, isoleucine, alpha tocopherol, and cholesterol are up-regulated in HCC vs. cirrhosis, while citric acid, lactic acid, and sorbose are down-regulated. Fig 3 depicts dot plots for valine, leucine, isoleucine, glutamic acid, and alpha tocopherol, showing an increase in metabolite levels from cirrhosis to Stage I HCC and progressing to Stages II & III, whereas citric acid, lactic acid, and sorbose are down-regulated in HCC vs. cirrhosis. To further evaluate the ability of these nine metabolites in distinguishing HCC cases from patients with liver cirrhosis, we performed both partial least squares discriminant analysis (PLS-DA) and orthogonal PLS-DA (OPLS-DA) using the metabolomic data from the targeted analysis. Fig 4A shows a score plot obtained by PLS-DA, which illustrates the separation between the HCC cases (red triangles) and patients with liver cirrhosis (blue dots). Stage II & III HCC cases are shown with solid triangles. Fig 4B depicts the corresponding loading plot, where the nine metabolites are highlighted. The figure demonstrates the ability of these metabolites in distinguishing HCC cases from cirrhotic controls. Also, the relevance of these metabolites is illustrated in Fig 4C through an S-plot obtained by OPLS-DA, where the nine metabolites stand out from all other metabolites considered in our targeted analysis.

Branched-chain amino acids (BCAAs), *i*.*e*. valine, leucine, and isoleucine, have been reported to have connections with several types of cancer including HCC. They have been also connected to other liver diseases such as cirrhosis [32]. However, the normal requirements for BCAAs are complicated by the conflicting needs of the tumor and the host. For example, BCAAs activate mammalian target of rapamycin (mTOR) signaling and enhance growth and proliferation of myocytes and epithelial cells, which is why BCAAs are increasingly used as a treatment for cancer cachexia. However, BCAA supplementation has also shown to significantly enhance pancreatic tumor growth via increased phosphorylation of mTOR and downstream effector S6 ribosomal protein in the same mTOR pathway [33].

The up-regulated BCAAs in our HCC samples may have tumorigenic effect in the liver. Thus, it will be important to test the effect of BCAAs in HCC models in future. We found valine, leucine, and isoleucine in our targeted analysis as significant (p-value < 0.05). However, in the untargeted analysis, only valine’s adjusted p-value was significant (q-value < 0.1). Valine was previously reported to have a significantly different level in HCC vs. normal controls in analysis of tissues by LC-MS [14] and serum samples by GC-MS [24].

Glutamine pathway, which includes glutamic acid, was previously reported as being upregulated in HCC vs. normal [14]. Also, glutamic acid transporter was reported to be overexpressed in HCC tissues compared to adjacent normal tissues in mRNA measurements [31]. Glutamine plays a role in upregulation of mTOR signalling pathway to facilitate proliferation in tumor cell growth and interestingly export of glutamine to the extracelular space via the L-type amino acid transportes such as LAT1, which is coupled with the import of essential amino acids such as BCAAs [34]. The BCAAs then function as described above to activate mTOR signalling and tumor cell proliferation.

As such sugars are not carcinogenic, however, among eight different sugars, sorbose showed mild sarcomas in rats [35]. It may be possible that coupled with overexpression of sorbose-specifc transporters, such as GLUT5 [36], sorbose may show higher carcinogenicity. While glucose is used in ribose production of proliferating cells through pentose phosphate pathway, it can be converted to citrate via glycolysis and Krebs cycle. Tumor cells may use citrate directly to fuel their metabolism and proliferation. Also citrate can be converted to Acetyl-CoA by ATP citrate lyase (ACL) and participate in the synthesis of fatty acids and cholesterol, which are essential components of cancer cell membranes, lipid raft and lipid-modified signalling molecules [37]. Notably, ACL knockdown can impair the Akt-meditated tumor growth *in vivo* [38]. Down-regulation of lactate as observed in our HCC samples may favor tumor growth. Lactate is reported to suppress proliferation, cytolytic activity of cytotoxic T lymphocytes (CTLs), and production of cytokine [39,40]. Finally, cholesterol has been suggested as a direct regulator of Akt-dependent signaling in prostate cancer cells linking to tumor cell survival which is functionally relevant to long-term advantages of cancer-preventive cholesterol-lowering drugs [41].

**Fig 5:**
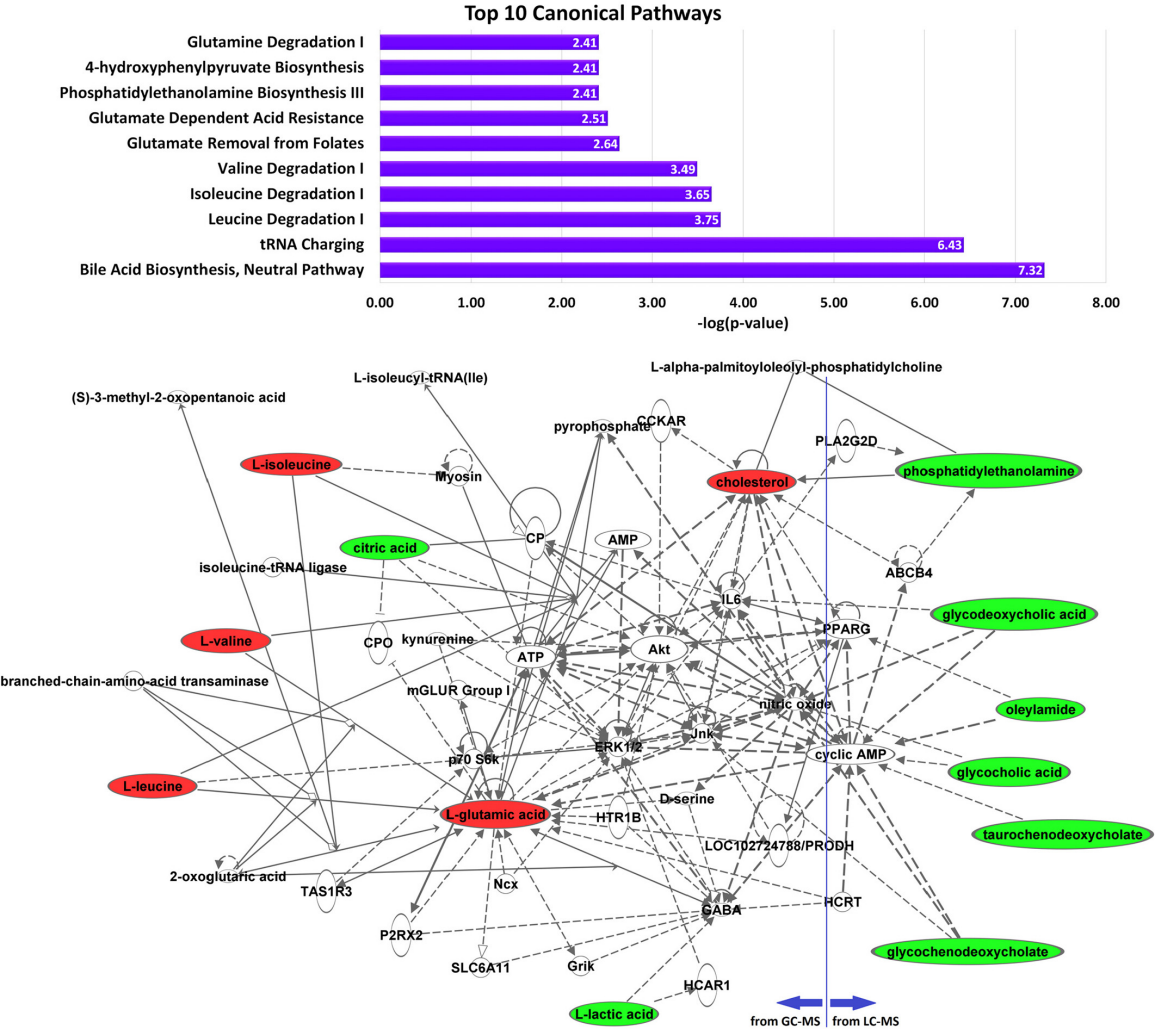
Pathway and network analysis of 24 metabolites recognized by IPA from the candidates discovered by GC-MS and LC-MS based analyses. A: top 10 canonical pathways based on 24 metabolites. B: network involving 13 out of the 24 metabolites (up-regulated in HCC vs. cirrhosis marked in red, down-regulated in HCC vs. cirrhosis marked in green).

To further investigate the relationship of candidate biomarkers to HCC, we performed pathway analysis using the Ingenuity Pathway Analysis (IPA) tool based on two sets of metabolites: (1) the nine metabolites that were found statistically significant in our GC-MS based study; (2) 15 metabolites previously reported in our LC-MS based metabolomic analysis of serum samples from the same subjects [18]. The pathway analysis reveals that glycochenodeoxycholate, glycocholic acid, and taurochenodeoxycholate from the LC-MS based study contribute to the enrichment of bile acid biosynthesis neutral pathway, whereas the metabolites from the GC-MS based study lead to the enrichment of several canonical pathways including tRNA charging, isoleucine degradation, valine degradation, glutamate dependent acid resistance, and glutamate degradation pathways. Fig 5A depicts the top 10 canonical pathways identified by IPA based on all metabolites from the three groups combined. Among these, IPA used 13 metabolites (6 from the LC-MS and 7 from the GC-MS based studies) to construct a network shown in Fig 5B. The 13 metabolites are known to be involved in lipid metabolism, molecular transport, and small molecule biochemistry. Fig 5B also shows that cyclic AMP (cAMP) and Akt, which are highly relevant in liver cancer, play a prominent role in the newtwork. cAMP activates cAMP-response element-binding (CREB) protein, a transcription factor, which is involved in cell proliferation, differentiation, cell-cycle progression, and cell survival acting as an oncogene [42,43]. CREB and phosphorylated form of CREB proteins have been shown to be significantly elevated in HCC versus normal liver and may be associated with tumor progression in HCC [44,45]. The Akt is a critical factor in mTOR signalling patway affecting HCC progression [30]. This further suggests that BCAAs and glutamic acid can be considered candidate biomarkers for liver cancer since they are known to activate Akt-driven mTOR pathway as described above.

## Conclusion

This paper focuses on identifying biomarkers for HCC by analysis of metabolites in plasma samples from participants recruited in Egypt. The levels of metabolites are evaluated in plasma samples from HCC cases and those from patients with liver cirrhosis using two GC-MS systems in an untargeted metabolomic analysis. The untargeted analysis leads to the identification of 27 metabolites that showed statistically significant differences between HCC cases and cirrhotic controls with false discovery rate less than 10%. These and other candidate metabolites (71 analytes in total) are further evaluated through targeted analysis by GC-SIM-MS. The targeted analysis confirms the significance of nine metabolites in distinguishing HCC cases from patients with liver cirrhosis. The candidate biomarkers include glutamic acid, alpha tocopherol, valine, isoleucine, leucine, and cholesterol that are up-regulated in HCC vs. cirrhosis, whereas citric acid, lactic acid, and sorbose are down-regulated. The results are complementary to our previous LC-MS based study on sera from the same cohort. We performed pathway analysis by combining the results from GC-MS- and LC-MS-based analyses. While candidate biomarkers discovered by our LC-MS based study are primarily involved in bile acid biosynthesis, those detected by GC-MS represent BCAA metabolism.

## Supporting Information

S1 Document Quality assessment. (PDF)

S1 Fig Confirmation of metabolites’ identities using standards. Identities of the following seven metabolites found to be significant in the targeted analyses were confirmed by the analysis of authentic compounds purchased from Sigma Aldrich: L-glutamic acid (95436), DL-alpha-tocopherol (47783), L-valine (PHR1172), L-(+)-lactic acid (46937), D-(+)-sorbose (S4887), DL-isoleucine (298689), and citric acid (94676). Individual 0.25 mg/mL stock standards solutions were prepared in appropriate solvent and stored at -20°C until the analysis. Working standards solutions, at the concentration of 1.25 μg/mL, were prepared by appropriate dilution of the stock standard solutions in acetonitrile, isopropanol, and water (3:3:2). Standards were then concentrated to dryness and derivatized following the same procedure as for the serum samples described in the material and method paragraph. Each standard was analyzed in both GC-qMS and GC-TOFMS platform, following the same GC and MS methods as previously described in the “Acquisition of GC-MS Data by Untargeted Method” section. Acquired spectra of the individual standards were cross matched with the corresponding spectra extracted from analysis of plasma samples. Representative spectra of the comparisons between the plasma metabolites and the standards are shown in Panels A-G.
(PDF)

S1 Table Experimental design. (XLSX)

S2 Table List of analytes used for targeted SIM analysis (complete SIM list). (XLSX)

S3 Table Spectra of statistically significant unidentified analytes from untargeted analysis. (XLSX)
