# Supplementary material for: GC-MS Based Plasma Metabolomics for Identification of Candidate Biomarkers for Hepatocellular Carcinoma in Egyptian Cohort
Source: PLoS One. 2015 Jun 1;10(6):e0127299. doi: 10.1371/journal.pone.0127299 (PMC4452085; doi:10.1371/journal.pone.0127299)
Supplement: S1 Document — (PDF) [file pone.0127299.s001.pdf]

Quality Assessment—Retention index (RI) standard mixture was spiked into each sample analyzed by GC- TOFMS. The standard was prepared by mixing a series of fatty acid methyl esters (FAMEs) with a series of alkanes. Specifically, FAMEs, were dissolved in chloroform at a concentrations of 0.8mg/mL (C8 – C16) and 0.4mg/ml (C18 – C30). 100µL of each FAME standard was mixed together and 1.2mL of chloroform was added for a final volume of 2.5mL (FAME). The Alkane standard comprised of all 16 evenly-numbered n-alkanes from C10 to C40, at a concentration of 50mg/L in n-heptane each. Alkane mixture was mixed with FAME at a ratio of 1:4, vortexed prior to spiking it into each sample. For the samples analyzed by GC-qMS, the RI standard mixture consisted of only FAMEs.

The blank samples were prepared together with the samples by adding the derivatization agent to an empty tube and following the same steps.

A pooled QC sample was analyzed in each batch by pooling 5 µL of each derivatized sample within the batch. The QC was run multiple times at the beginning of each batch, in between runs, and at the end of each batch. The QCs at the beginning were used for column conditioning. The data from the remaining QC runs were used for quality assessment. In both cases, 60µL of the mixed reaction mixture was transferred into 250µL clear glass autosampler vials with low-volume inserts and cap.

QC samples were used to investigate the consistency and reproducibility of the peaks across different runs and batches. Also six spiked deuterated internal standards (ISs), i.e., myristic acid d27, phenylalanine-phenyl-d8, alanine-d4, tyrosine-d2, glycine-d5 and glutamic acid-d5, were used for quality assessment based on their variability across different runs. In addition, outlier screening is performed to remove runs that are significantly different from the others in terms of

the number of detected peaks or the chromatographic pattern. Also, we filtered the peaks based on the number of missing values per group. Specifically, peaks that were not detected in at least 50% of the samples in either the case or control group were removed from the feature list. The remaining peaks were analyzed to identify statistically significant changes in metabolite levels between the cases and controls.

Also, in the targeted analysis by GCqMS, we performed RI calibration using FAME. Multiple injections of a pooled QC samples were run for equilibrium and a pooled case sample and a pooled control sample were also run in the full scan mode for analyte verification and RT confirmation.
