# Supplementary material for: GC-MS Based Plasma Metabolomics for Identification of Candidate Biomarkers for Hepatocellular Carcinoma in Egyptian Cohort
Source: PLoS One. 2015 Jun 1;10(6):e0127299. doi: 10.1371/journal.pone.0127299 (PMC4452085; doi:10.1371/journal.pone.0127299)
Supplement: S1 Fig — Identities of the following seven metabolites found to be significant in the targeted analyses were confirmed by the analysis of authentic compounds purchased from Sigma Aldrich: L-glutamic acid (95436), DL-alpha-tocopherol (47783), L-valine (PHR1172), L-(+)-lactic acid (46937), D-(+)-sorbose (S4887), DL-isoleucine (298689), and citric acid (94676). Individual 0.25 mg/mL stock standards solutions were prepared in appropriate solvent and stored at -20°C until the analysis. Working standards solutions, at the concentration of 1.25 μg/mL, were prepared by appropriate dilution of the stock standard solutions in acetonitrile, isopropanol, and water (3:3:2). Standards were then concentrated to dryness and derivatized following the same procedure as for the serum samples described in the material and method paragraph. Each standard was analyzed in both GC-qMS and GC-TOFMS platform, following the same GC and MS methods as previously described in the “Acquisition of GC-MS Data by Untargeted Method” section. Acquired spectra of the individual standards were cross matched with the corresponding spectra extracted from analysis of plasma samples. Representative spectra of the comparisons between the plasma metabolites and the standards are shown in Panels A-G. (PDF) [file pone.0127299.s002.pdf]

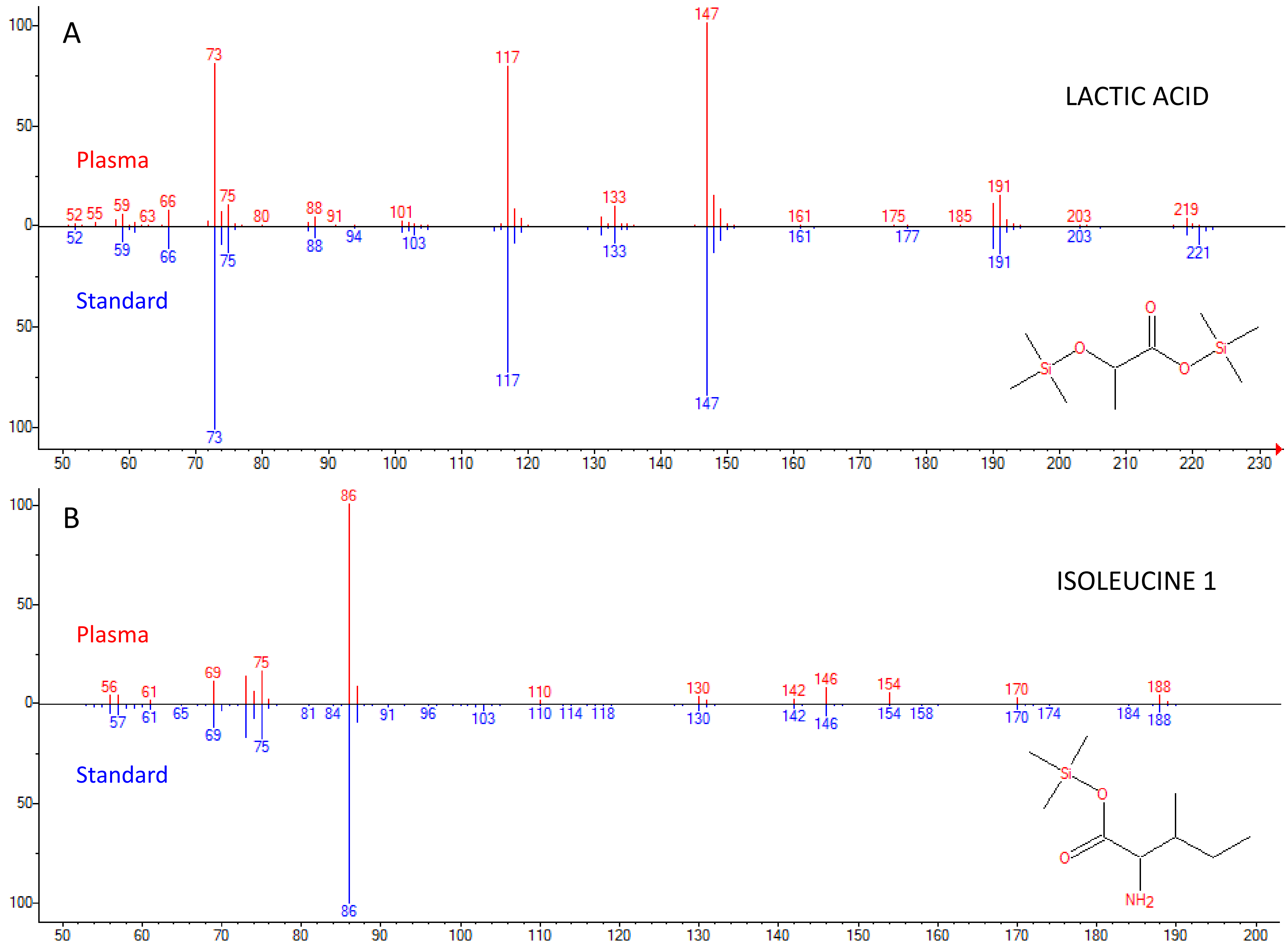
A
LACTIC ACID
Plasma
Standard
B
ISOLEUCINE 1
Plasma
Standard
Si
O
NH2

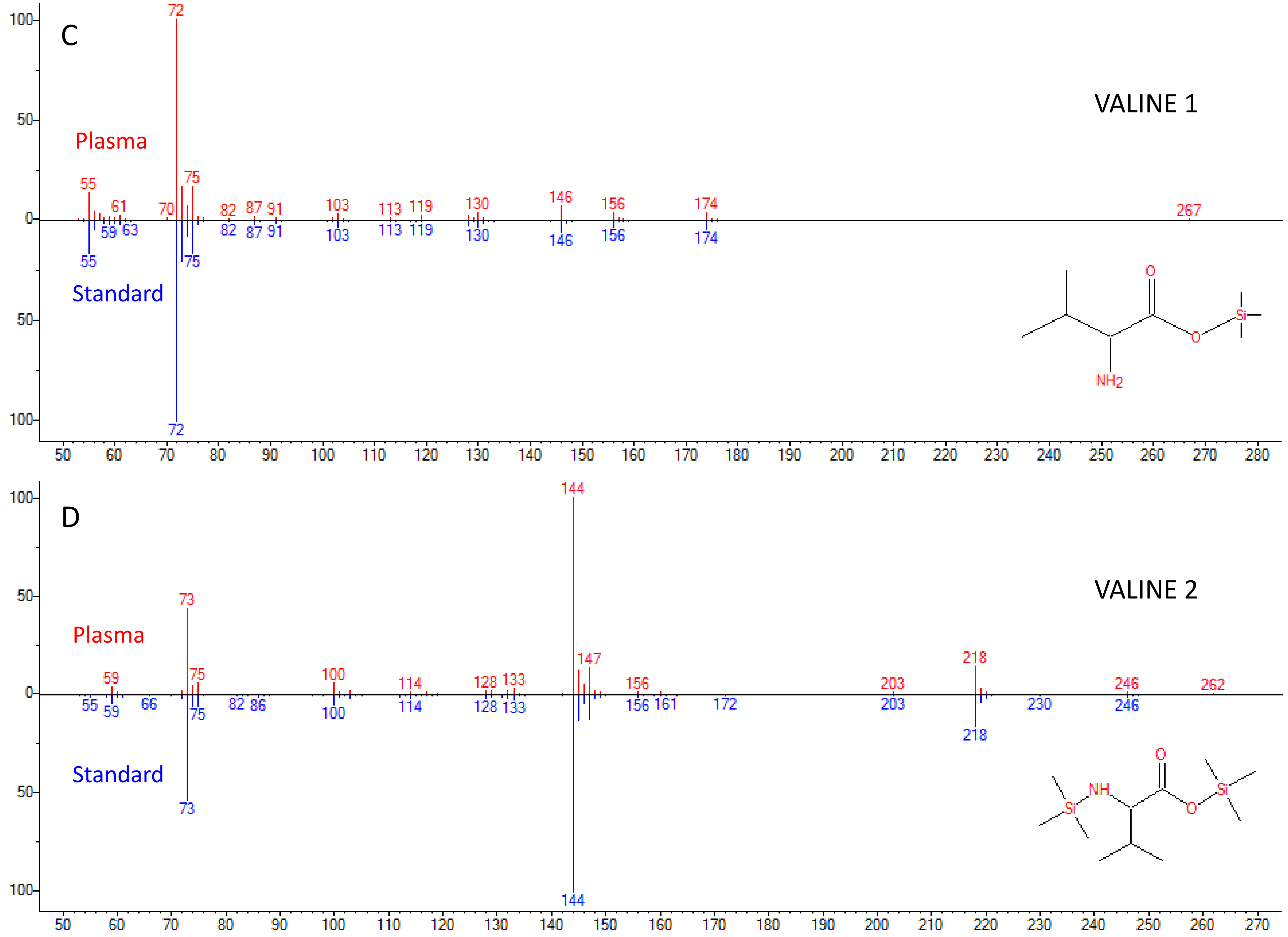
C
VALINE 1
Plasma
Standard
72
55
61
70
75
82 87 91
103
113 119
130
146
156
174
267
59 63
NH2
O
Si
D
VALINE 2
Plasma
Standard
144
73
59
75
100
114
128 133
147
156
203
218
246
262
55 59 66
82 86
161
172
230
NH
O
Si

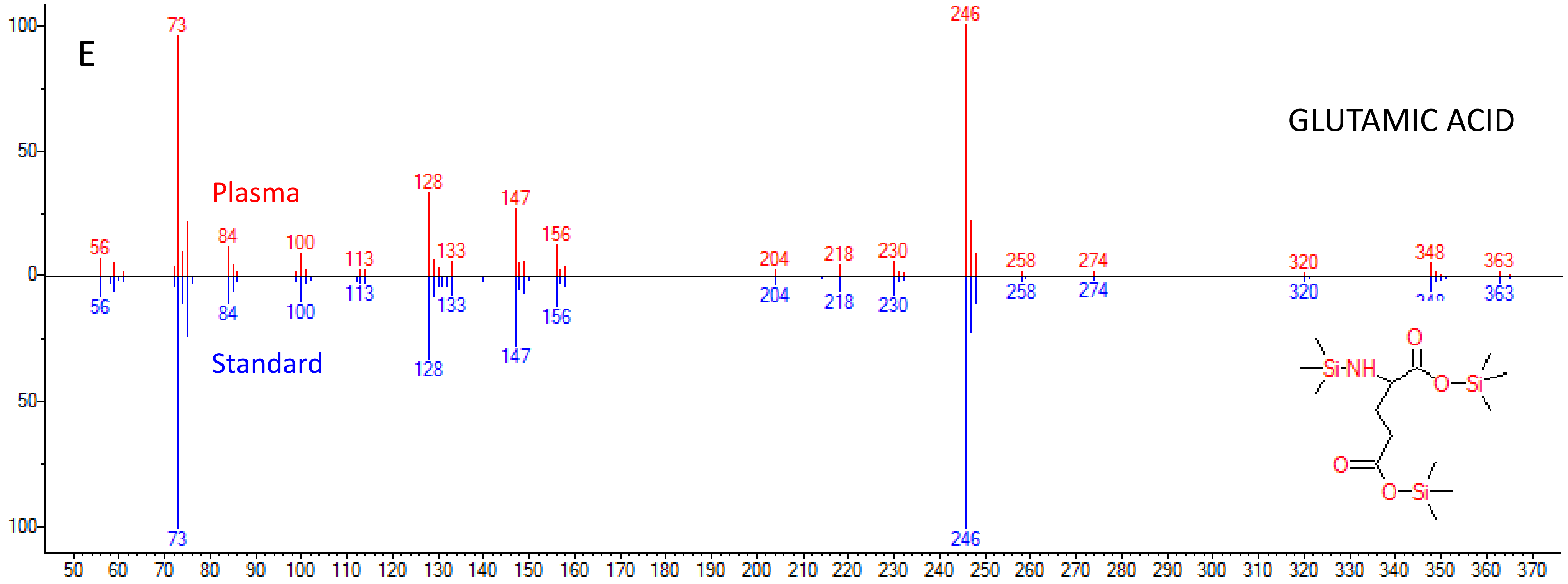
E
GLUTAMIC ACID
Plasma
Standard
73
246
128
147
156
56
84
100
113
133
204
218
230
258
274
320
348
363
Si-NH
O
O-Si
O-Si

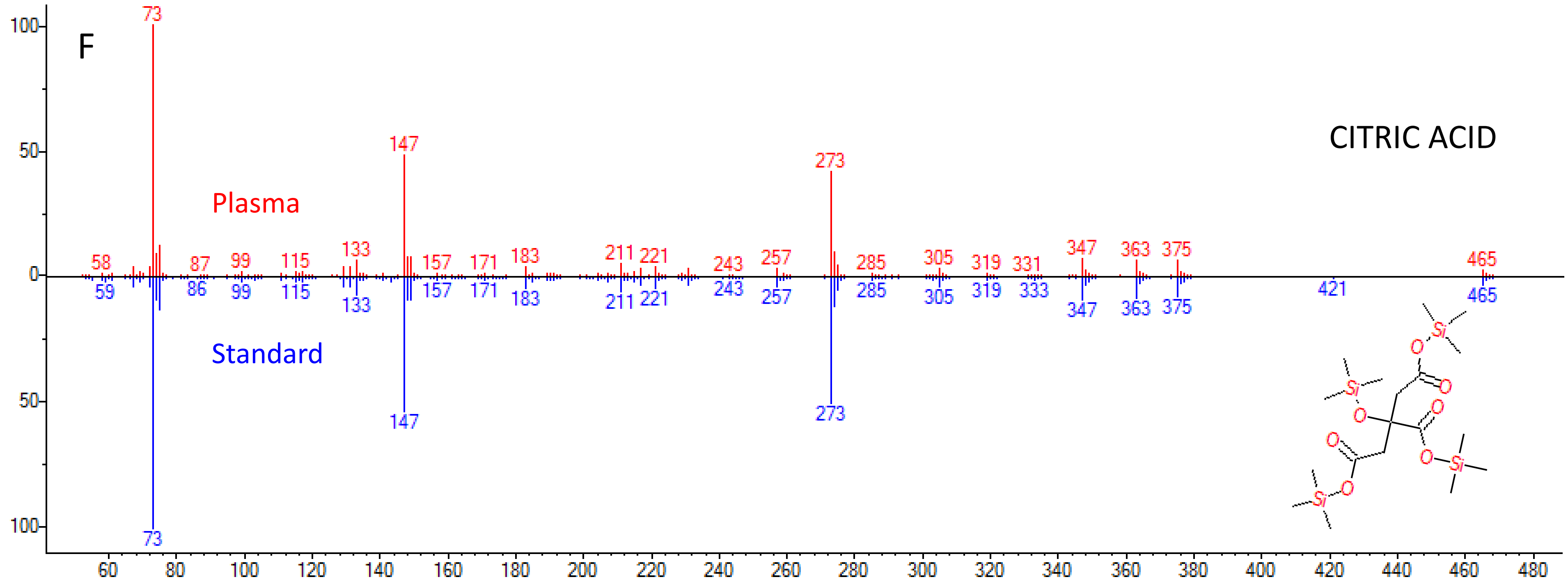
F
CITRIC ACID
Plasma
Standard
73
147
273
58
59
87
86
99
115
133
157
171
183
211
221
243
257
285
305
319
331
333
347
363
375
421
465
Si
O

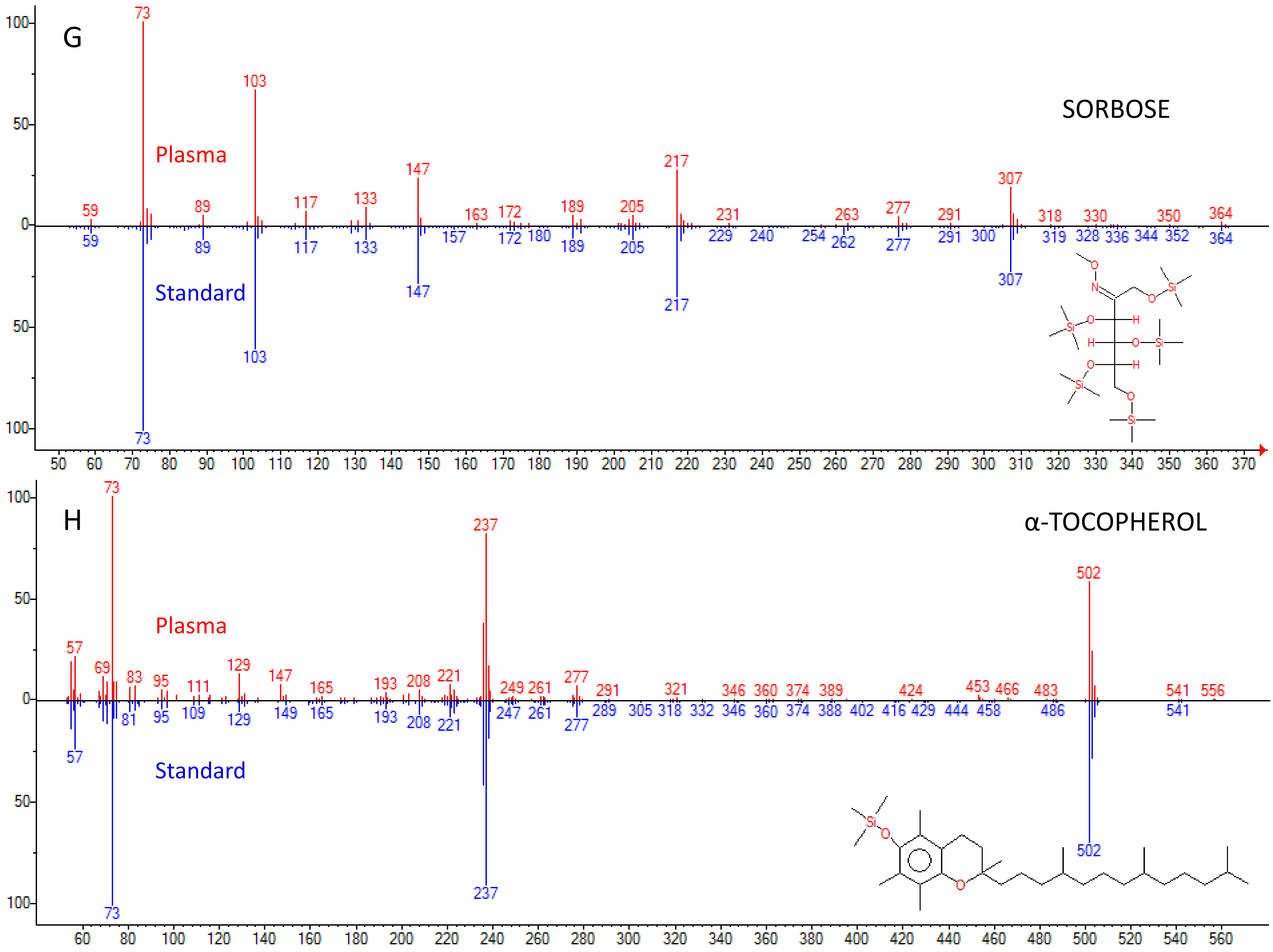
G
SORBOSE
Plasma
Standard
73 103 147 217 307
59 89 117 133 163 172 189 205 231 263 277 291 318 330 350 364
59 89 117 133 157 172 180 189 205 229 240 254 262 277 291 300 319 328 336 344 352 364
50 60 70 80 90 100 110 120 130 140 150 160 170 180 190 200 210 220 230 240 250 260 270 280 290 300 310 320 330 340 350 360 370
H
α-TOCOPHEROL
Plasma
Standard
73 237 502
57 69 83 95 111 129 147 165 193 208 221 249 261 277 291 321 346 360 374 389 424 453 466 483 541 556
57 81 95 109 129 149 165 193 208 221 247 261 277 289 305 318 332 346 360 374 388 402 416 429 444 458 486 541
60 80 100 120 140 160 180 200 220 240 260 280 300 320 340 360 380 400 420 440 460 480 500 520 540 560

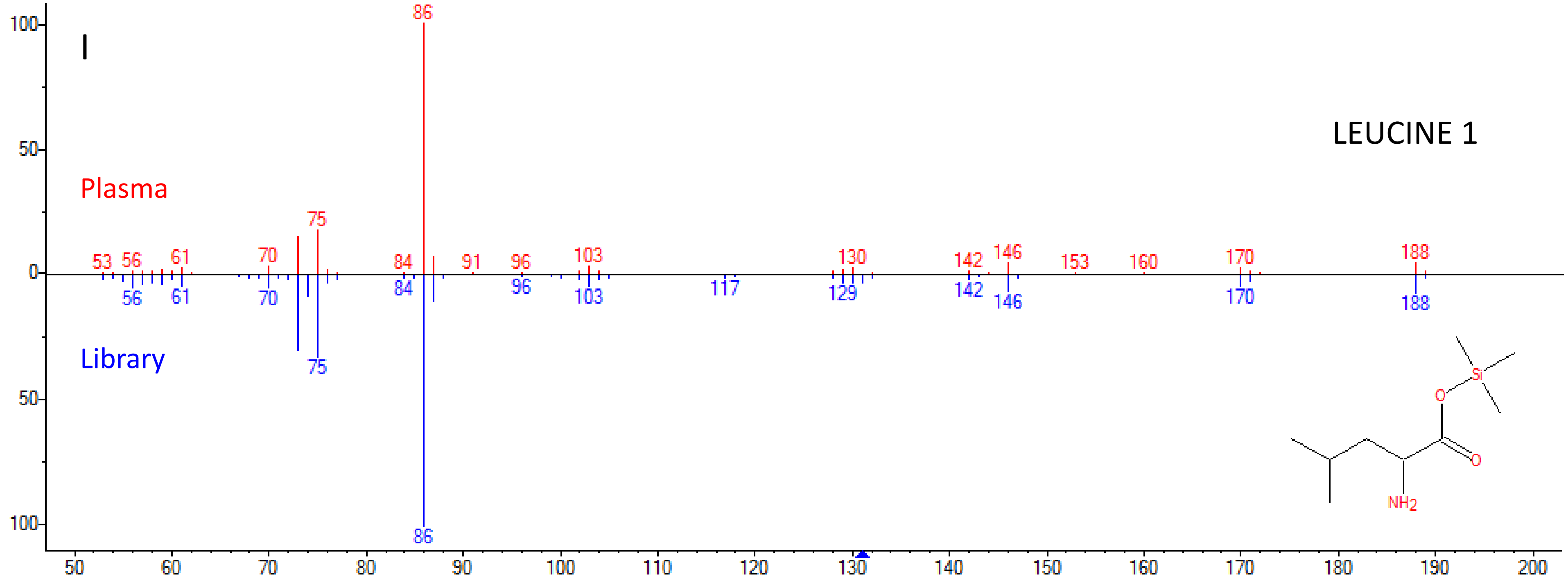
I
LEUCINE 1
Plasma
Library
86
75
53 56 61 70 84 91 96 103 130 142 146 153 160 170 188
56 61 70 84 96 103 117 129 142 146 170 188
O
Si
O
NH2
100
50
0
50
100
50 60 70 80 90 100 110 120 130 140 150 160 170 180 190 200

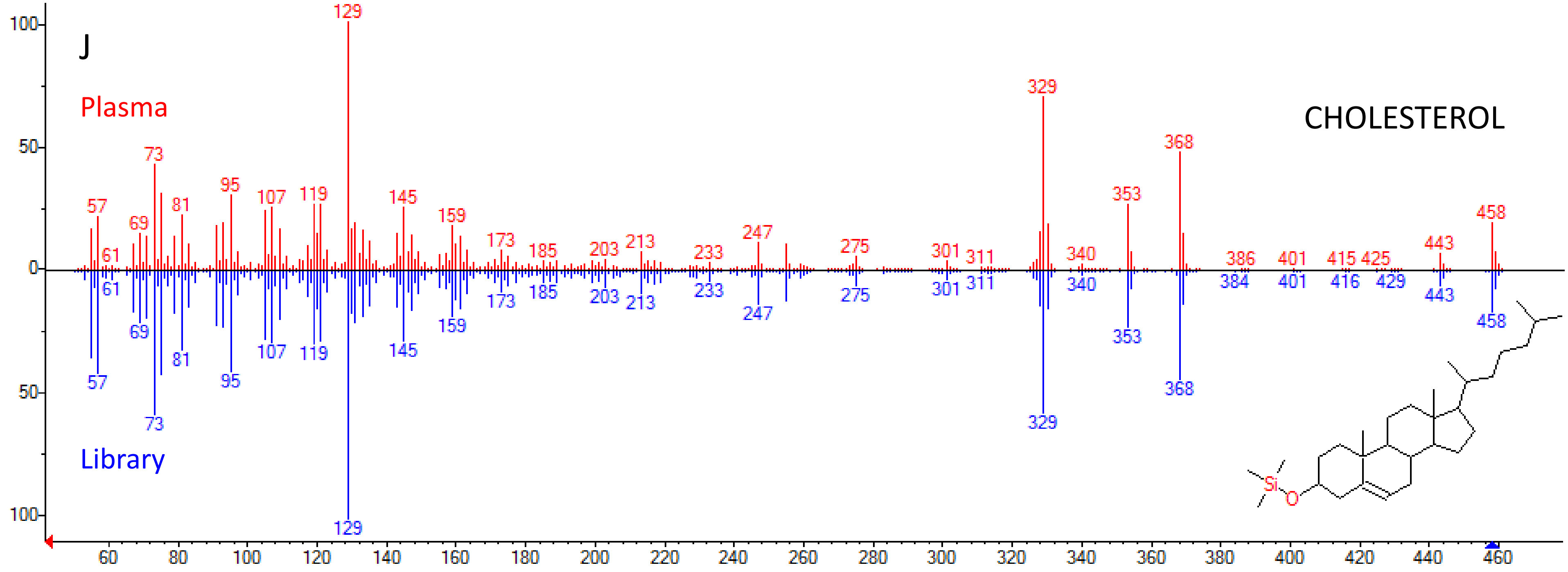
J
CHOLESTEROL
Plasma
Library
129
329
368
353
458
57 61 69 73 81 95 107 119 145 159 173 185 203 213 233 247 275 301 311 340 386 401 415 425 443
57 61 69 73 81 95 107 119 129 145 159 173 185 203 213 233 247 275 301 311 329 340 353 368 384 401 416 429 443 458
Si
O
100
50
0
50
100
60 80 100 120 140 160 180 200 220 240 260 280 300 320 340 360 380 400 420 440 460

Figure S1. Confirmation of metabolites' identities using standards. Identities of the following seven metabolites found to be significant in the targeted analyses were confirmed by the analysis of authentic compounds purchased from Sigma Aldrich: L-glutamic acid (95436), DL-alfa-tocopherol (47783), L-valine (PHR1172), L-(+)-lactic acid (46937), D-(+)-sorbose (S4887), DL-isoleucine (298689), and citric acid (94676). Individual 0.25 mg/mL stock standards solutions were prepared in appropriate solvent and stored at -20 °C until the analysis. Working standards solutions, at the concentration of 1.25 µg/mL, were prepared by appropriate dilution of the stock standard solutions in acetonitrile, isopropanol, and water (3:3:2). Standards were then concentrated to dryness and derivatized following the same procedure as for the serum samples described in the material and method paragraph. Each standard was analyzed in both GCq and GC-TOF platform, following the same GC and MS methods as previously described in the "Acquisition of GC-MS Data by Untargeted Method" section. Acquired spectra of the individual standards were cross matched with the corresponding spectra extracted from analysis of plasma samples. Representative spectra of the comparisons between the plasma metabolites and the standards are shown in Panels A-G. For two metabolites (leucine 1 and cholesterol), the comparisons were made between spectra from plasma samples and reference spectra extracted from the Fiehn library as illustrated in Panels H & I.
